# Supplementary material for: The Association of ADAMTS7 Gene Polymorphisms with the Risk of Coronary Artery Disease Occurrence and Cardiovascular Survival in the Polish Population: A Case-Control and a Prospective Cohort Study
Source: Int J Mol Sci. 2024 Feb 14;25(4):2274. doi: 10.3390/ijms25042274 (PMC10889572; doi:10.3390/ijms25042274)
Supplement: Supplementary file 1 [file ijms-25-02274-s001.zip › ijms-2849808-supplementary.pdf]

Table S1. Synergistic effect of the *ADAMTS7* gene polymorphisms and elevated parameters of lipid metabolism.

rs3825807 A allele	TC ≥ 5 mmol/L	CAD (n =212)	Controls (n = 233)	OR (95% CI)
1	1	130	81	3.39 (1.46 – 7.85)
1	0	54	103	1.11 (0.47 – 2.61)
0	1	19	30	1.34 (0.50 – 3.56)
0	0	9	19	1
rs3825807 A allele	LDL ≥ 3 mmol/L	CAD (n =210)	Controls (n = 233)	OR (95% CI)
1	1	147	86	3.63 (1.50 – 8.77)
1	0	35	98	0.76 (0.30 – 1.91)
0	1	20	32	1.33 (0.48 – 3.64)
0	0	8	17	1
rs1994016 C allele	TC ≥ 5 mmol/L	CAD (n =211)	Controls (n = 232)	OR (95% CI)
1	1	131	81	3.05 (1.30 – 7.18)
1	0	53	104	0.96 (0.40 – 2.30)
0	1	18	30	1.13 (0.42 – 3.07)
0	0	9	17	1
rs7173743 T allele	TC ≥ 5 mmol/L	CAD (n =212)	Controls (n = 233)	OR (95% CI)
1	1	125	77	2.84 (1.32 – 6.10)
1	0	51	101	0.88 (0.40 – 1.98)
0	1	24	34	1.24 (0.51 – 2.98)
0	0	12	21	1

TC – total cholesterol, LDL – low-density lipoprotein, OR – Odds Ratio, CAD – coronary artery disease (patient group).
